# Evaluation of precipitation across the contiguous United States, Alaska, and Puerto Rico in multi-decadal convection-permitting simulations

**DOI:** 10.1038/s41598-024-51714-3

**Published:** 2024-01-12

**Authors:** Akintomide Afolayan Akinsanola, Chunyong Jung, Jiali Wang, Veerabhadra Rao Kotamarthi

**Affiliations:** 1 Environmental Science Division, Argonne National Laboratory, 9700 South Cass Avenue, Building 240, Lemont, IL 60439 USA; 2 Department of Earth and Environmental Sciences, University of Illinois Chicago, Chicago, IL USA

**Keywords:** Atmospheric science, Environmental sciences

## Abstract

This study is an early effort to generate a multi-decadal convection-permitting regional climate dataset that covers nearly the entire North American continent. We assessed a 20 year dynamically downscaled regional climate simulation at a 4 km spatial resolution with explicit convection across the contiguous United States (CONUS), Alaska, and Puerto Rico. Specifically, we evaluated the model’s performance in representing mean, 95th percentile, and extreme precipitation across regions. Our findings indicate that when compared with ERA5 reanalysis, the forcing data, convection-permitting simulation improves representations of seasonal, 95th percentile, and extreme precipitation over a large portion of the CONUS, Alaska, and Puerto Rico, particularly in areas where precipitation is heaviest. The simulation adds value over its forcing data (ERA5) in up to 53% of all grid cells in the CONUS, 68.8% in Alaska, and 84.0% in Puerto Rico. It is important to note that, however, despite improvements, model errors in Puerto Rico remain large. Similar improvements are observed in extreme indices, including consecutive dry days, maximum 5 days precipitation, and extreme precipitation. Analysis of the diurnal cycle of mean hourly precipitation suggests that representations of convective processes—including onset, dissipation, suppression, downstream propagation, and local circulation—improved overall.

## Introduction

Precipitation significantly affects many sectors of society and the environment, and understanding it is crucial to addressing environmental, social, and economic issues. Accurately representing precipitation in numerical models is essential for assessing potential climate change impacts, including rainfed agriculture, water resource management, and hydroelectric power generation^1–5^.

However, typical general circulation models (GCMs) with coarse resolution (60–300 km) cannot resolve the small-scale processes of convection or complex terrain features, which limits their ability to provide detailed information at regional and local scales. To address these limitations, downscaling techniques^6–10^ have been developed over the years to bridge the gap between the climate scale at which synoptic climatology is studied and the scale necessary for regional or local assessment. One of these approaches is called dynamical downscaling; this involves using GCMs or reanalysis to provide the initial and lateral boundary conditions for regional climate models (RCMs). RCM simulations, conducted at high resolutions over specific regions, typically excel in resolving clouds, orography, coastal zones, land use/land cover effects, and local-scale circulations that are often beyond the capability of GCMs^11–17^.

Increasing the horizontal resolution at which an RCM can explicitly resolve convection (~ 4 km; convection-permitting [CP] resolution) is becoming more common. At this resolution and finer, cumulus parameterizations can be switched off, enabling a large part of atmospheric deep convection to be explicitly resolved. Recent advances in computer capacity have led to more studies running RCM simulations at CP scales, and representations of precipitation have greatly improved across many regions^18–27^. In particular, several CP-scale RCM simulations have been conducted over the contiguous United States (CONUS) in recent years (e.g.,^25–27^), showing potential in accurately representing precipitation systems and processes and providing fine-scale climate datasets. However, these simulations were limited in geographic coverage and/or temporal length/resolution. As a result, they may not fully capture some high-impact weather events in which atmosphere–ocean interaction is important, such as tropical cyclones and atmospheric rivers. In addition, regions beyond the CONUS, such as Alaska and Puerto Rico, are underrepresented in both model simulations and observations; only a limited number of high-resolution gridded observation-based datasets are available for them (e.g.,^28,29^). Consequently, capacity to assess regional climate statistics, study long-term trends, explore local-dependent weather regimes, and provide valuable insights into climate extremes and risk assessments remains constrained.

**Figure 1:**
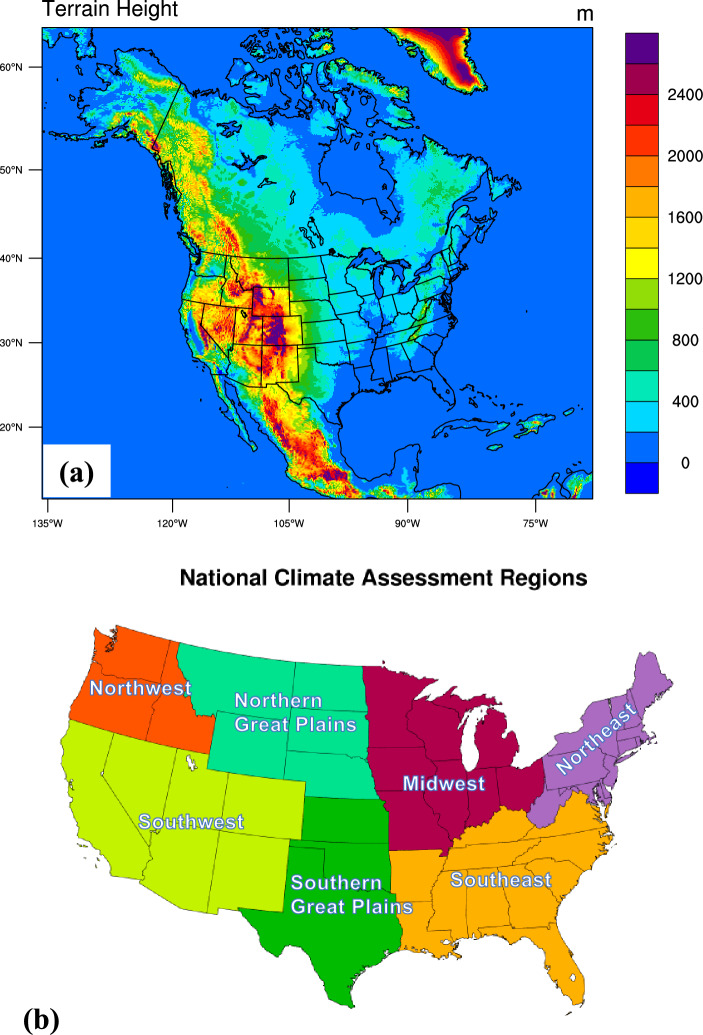
(**a**) WRF model domain with terrain height elevations (in meters) and (**b**) seven U.S. subregions defined by the fourth National Climate Assessment.

Our study builds on previous efforts in order to produce an hourly dataset spanning a 20-year period (2001–2020) at the CP scale. Specifically, our simulation domain covers nearly all of North America and a large portion of the North Atlantic and Eastern North Pacific Oceans, including Alaska, Mexico, and neighboring Caribbean islands, such as Puerto Rico (Fig. 1a). Note that we use a series of 14-month runs with 20 reinitializations rather than a continuous run (see Methods section for details). This new dataset is referred to as Argonne Dynamic Downscaled Achieve V2 (ADDA_V2).

It is important to include all these regions because climate change manifests differently across geographic areas. For example, Alaska has experienced a warming trend over the past decades that is more than twice as rapid as that over the CONUS^30^. Puerto Rico exhibits vast spatial variability, particularly in multi-decadal precipitation trends across the island, which may be due to its complex terrain and heterogeneity^31^. Furthermore, our extended coverage of the North Atlantic and Eastern North Pacific ocean basins will enable researchers to study high-impact weather systems and phenomena, including atmospheric rivers, tropical and extratropical cyclones, and precipitation associated with moisture transport from the Gulf of Mexico.

The objective of this study is to evaluate the performance of an RCM explicitly resolving convection at a very high resolution (4 km) in simulating precipitation characteristics, including mean and 95th percentile precipitation, and climate extreme indices (Table 1). This study also examines the multi-decadal CP simulation’s ability to represent diurnal precipitation patterns and associated convective processes through hourly mean precipitation (e.g., intensity, duration, timing, downstream propagation). Throughout the investigation, we explore the model bias compared to high-resolution gridded observations, such as PRISM and Daymet. We also highlight the potential added value (AV) of CP simulation compared to its driving data (European Centre for Medium-Range Forecast Reanalysis v5 [ERA5] reanalysis^32^) over the CONUS, Alaska, and Puerto Rico. This analysis provides valuable insights for qualitatively evaluating model performance and detecting model biases when compared to its forcing data (ERA5) and observations. It helps identify regions, seasons, and variables where the model excels, enabling users to make informed decisions about when and where to rely on the model’s output.

**Table 1:** Precipitation extreme indices used in this study.

No.	Extreme indices	Name	Definition	Units
1	CDD	Consecutive dry days	*PR*_*ij*_ is the daily precipitation amount on day *i* in period *j*. Count the largest number of consecutive days where *PR*_*ij*_ < 1 mm	days
2	RX5 day	Maximum consecutive 5-day precipitation	*PR*_*kj*_ is the precipitation amount for the 5-day interval ending *k*, period *j*. Then maximum 5 d values for period *j* are: RX5day_j_ = max (*PR*_*kj*_)	mm
3	R20 mm	Very heavy precipitation days	PR_ij_ is the daily precipitation amount on day *i* in period *j*. Count the number of days where PR_ij_ > 20 mm	days

## Results

### Seasonal mean daily precipitation

**Figure 2:**
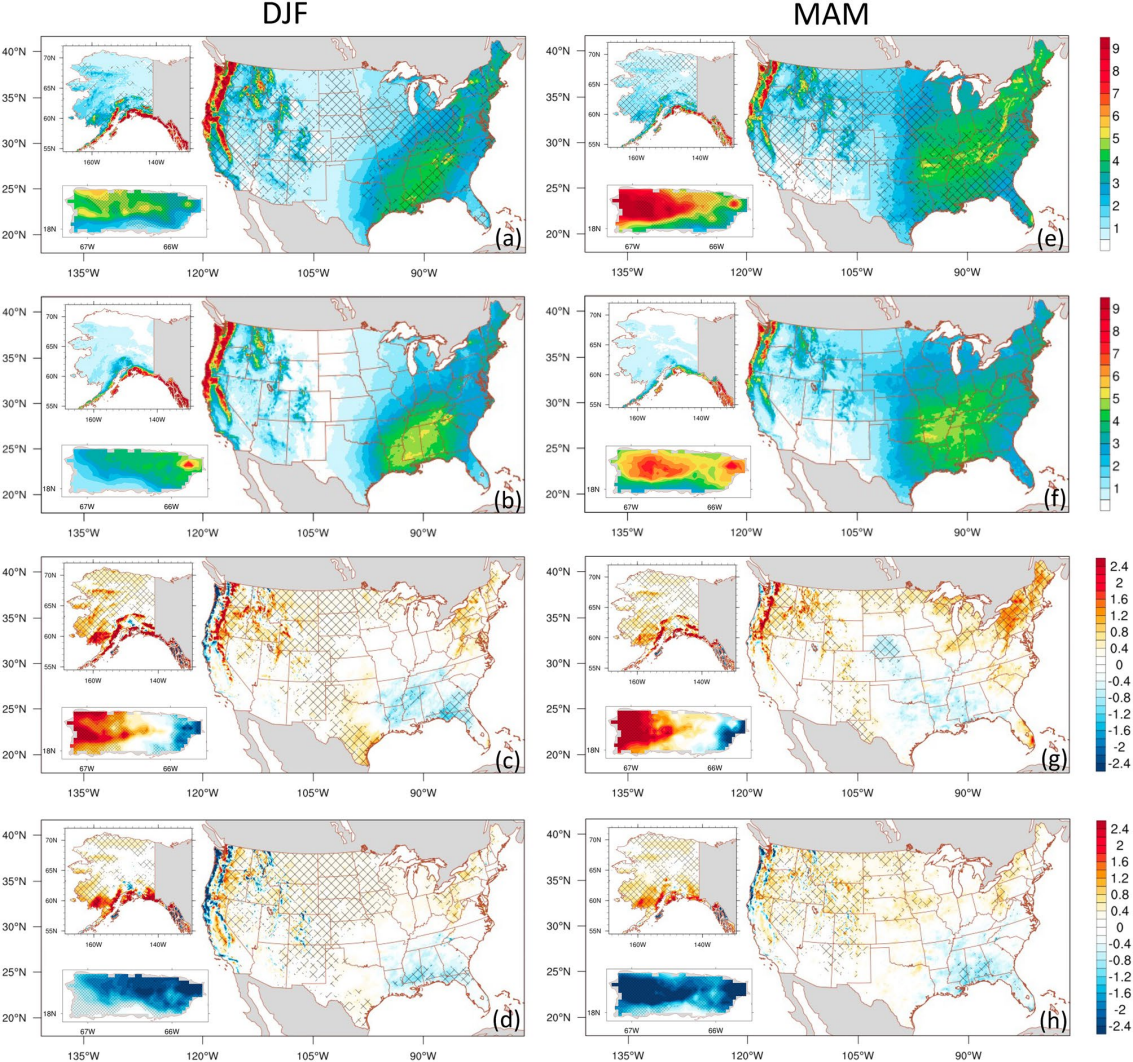

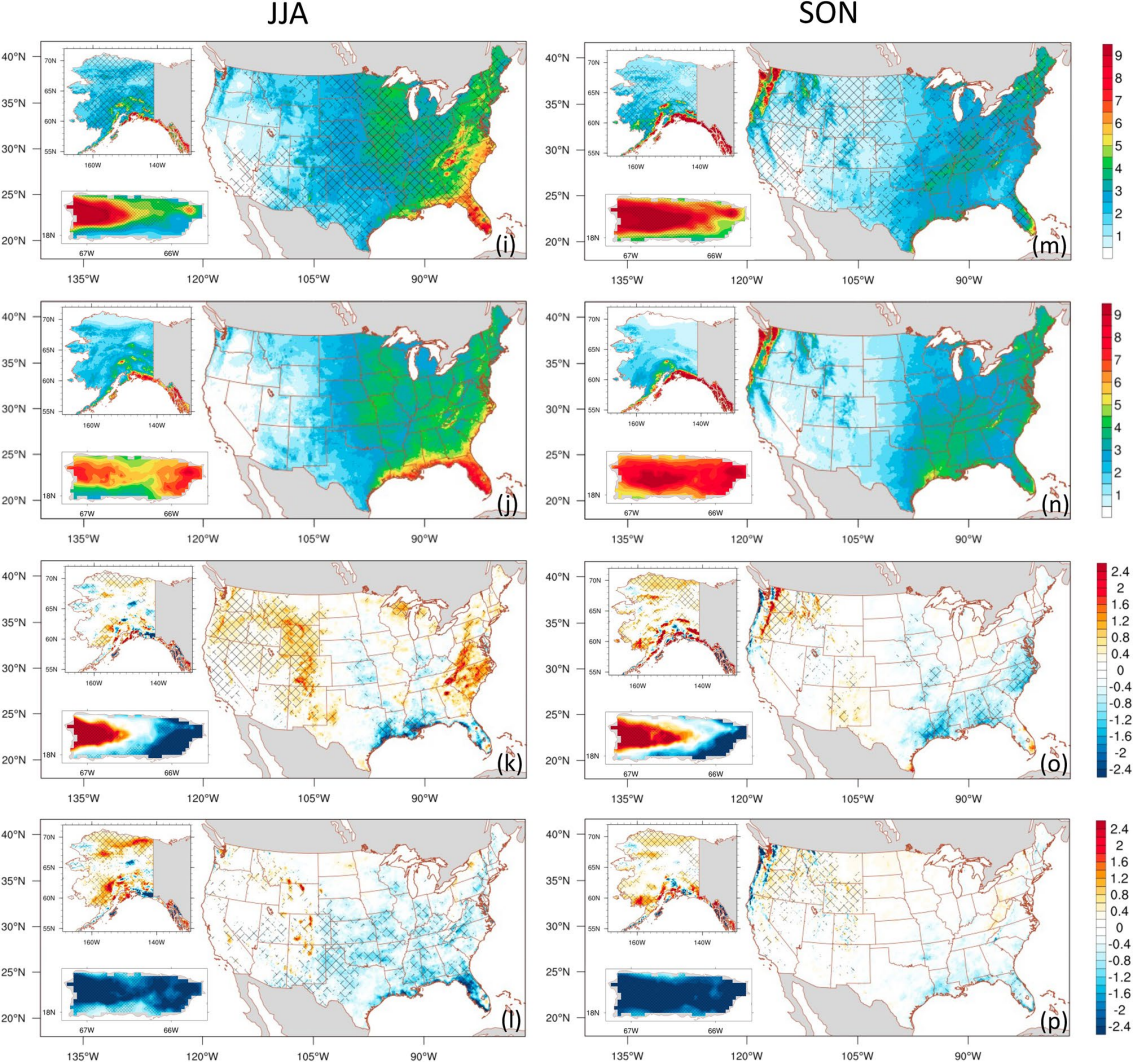
Spatial distribution of seasonal mean daily precipitation (mm day^−1^) for the CONUS, Alaska, and Puerto Rico during the 2001–2020 period. The data is sourced from ADDA_V2 (first row), Observation-based gridded dataset (second row), ADDA_V2 minus Observation-based gridded dataset (third row), and ERA5 minus Observation-based gridded dataset (fourth row). PRISM (Daymet) is utilized for CONUS (Alaska and Puerto Rico) as the observation-based gridded dataset. Hatches on the first row indicate grid points with value added by dynamical downscaling. On the third and fourth rows, grid points with statistically significant differences at 95% confidence level are marked with hatches.

We evaluated the ability of ADDA_V2, driven by ERA5 reanalysis, to reproduce seasonal mean daily precipitation over the CONUS, Alaska, and Puerto Rico (see Fig. 2). Precipitation simulated by ADDA_V2 over the CONUS is evaluated with PRISM^33^ (Precipitation-Elevation Regressions on Independent Slopes Model); that over Alaska and Puerto Rico is evaluated using Daymet V4^34^ (Daily Meteorological Surface Data).

During winter over the CONUS (Fig. 2b), the maximum (minimum) precipitation occurs in the northwestern and southeastern CONUS (the northern and southern Great Plains). An intense precipitation center in the northwest decreases significantly during the transition to spring (Fig. 2b,f), and there is evidence of the northward advancement of the precipitation band from the southeast.

In summer (Fig. 2j), the eastern half of the CONUS experiences high precipitation (> 4 mm day^−1^). Maximum precipitation occurs over Florida and the Gulf Coast, while both the northwest and southwest are dry (< 1 mm day^−1^). Intense precipitation occurs in the northwest in the fall (Fig. 2n), with moderate precipitation in the southeast.

Over Alaska, seasonal precipitation generally exhibits a north–south gradient, with pronounced precipitation peaks during fall and winter over southern Alaska. Similarly, Puerto Rico exhibits a meridional gradient of precipitation that is strongly related to regional orography. During winter, the entire island experiences significantly drier conditions compared to other seasons; fall is its wettest season.

**Figure 3:**
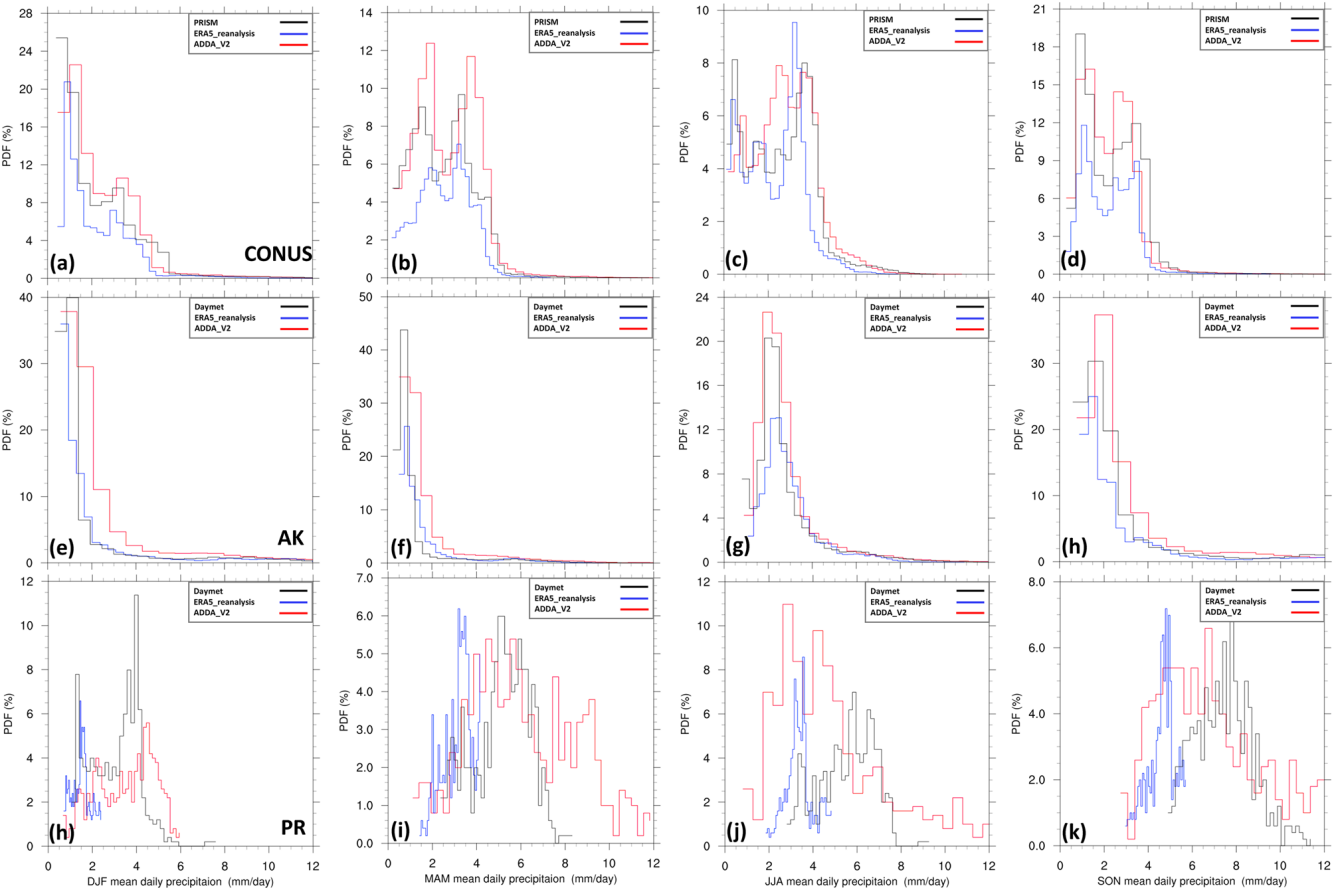
Probability density function of the seasonal mean daily precipitation (mm day^−1^) for the CONUS (first row), Alaska (AK, middle row), and Puerto Rico (PR, bottom row) for DJF (first column), MAM (second column), JJA (third column), and SON (fourth column). The data is derived from observations (black lines), ADDA_V2 (red lines), and ERA5 reanalysis (blue lines) averaged over the CONUS for the period of 2001–2020.

ERA5 reanalysis (Fig. 2d,h,l,p) does a relatively good job capturing the spatial distribution of seasonal mean daily precipitation observed in PRISM and Daymet; however, there is a pronounced bias across the three regions (i.e., CONUS, Alaska, Puerto Rico). Specifically, over the CONUS, it underestimates summer precipitation in the eastern half, showing a maximum dry bias of − 3.2 mm day^−1^ on the West Coast of Florida. It slightly overestimates winter and spring precipitation over the northern Great Plains, Midwest, and parts of the Northeast while underestimation is prevalent in the Southeast and West Coast. However, the bias is relatively low in fall (Fig. 2m–p). ERA5 grossly overestimates daily precipitation across all seasons in most of Alaska, with up to 38.1% more spatial-averaged rain in MAM; the overestimation is particularly pronounced in southern Alaska. Furthermore, in Puerto Rico, ERA5 is not capable of capturing details in orographic rainfall due to its coarse resolution; it significantly underestimates daily precipitation over all of Puerto Rico across all seasons, with a maximum spatial-averaged seasonal mean rain deficiency of 50.5% observed during DJF. ADDA_V2 (Figs. 2; first and third row) generally does a better job representing high-precipitation centers in most seasons across the three regions. This improvement, compared with ERA5, is particularly evident in the hatched areas across all seasons in the top row of Figs. 2 and 3. However, ADDA_V2 overestimates (underestimates) CONUS winter and summer precipitation over the northern and southern Great Plains, as well as spring precipitation over the northwestern and northeastern (fall precipitation over the southwestern) CONUS. These biases in ADDA_V2 are sometimes larger than those in ERA5 (for example, fall in the Southeast and summer in the Rockies). Nevertheless, based on AV analysis, ADDA_V2 shows improvement compared to ERA5 (Fig. 2a,e,i,m); hatched areas, indicating grid points with value added by dynamical downscaling, cover 46.9%, 48.2%, 46.7%, and 43.0% of all grid points in the CONUS in winter, spring, summer, and fall, respectively.

Over Alaska, ADDA_V2 captures pronounced peaks in seasonal precipitation, primarily in southern Alaska, better than ERA5 (Fig. 2a,e,i,m; hatched areas). In addition, ADDA_V2 mostly alleviates the biases evident across all seasons in ERA5, which improves representations of daily precipitation; AVs are 24.1%, 55.1%, 68.8%, and 42.4% across all grid points during winter, spring, summer, and fall, respectively.

Similar improvements by ADDA_V2 over the driving ERA5 are also evident over Puerto Rico. Although ADDA_V2 notably overestimates (underestimates) in the western (eastern) side of the island, it considerably mitigates the biases observed in ERA5 across all seasons in the region. There, AVs are 55.3%, 61.8%, 51.7%, and 75.1% during the winter, spring, summer, and fall, respectively (Fig. 2a,e,i,m).

Table 2 shows additional information on how ADDA_V2 performs compared to ERA5 and observations. Over the CONUS, the Taylor Skill Score (TSS) during winter, spring, summer, and fall is 0.951, 0.977, 0.972, and 0.968 for ERA5 and 0.995, 0.999, 0.995, and 0.986 for ADDA_V2. In Alaska, ADDA_V2 outperforms ERA5 in all seasons except winter. Conversely, ADDA_V2 exhibits lower scores than ERA5 in all seasons except winter in Puerto Rico.

**Table 2:** Descriptive statistics for daily mean seasonal precipitation.*

Precipitation (mm day^−1^)		DJF (CONUS/AK/PR)	MAM (CONUS/AK/PR)	JJA (CONUS/AK/PR)	SON (CONUS/AK/PR)
Standard deviation	Obs	1.749/2.610/1.148	1.328/1.544/1.260	1.469/1.544/1.266	1.314/3.174/1.293
	ADDA_V2	1.624/2.952/1.309	1.370/1.506/2.461	1.367/1.506/3.413	1.168/3.128/2.906
	ERA5	1.398/2.586/0.432	1.138/1.259/0.652	1.241/1.259/0.650	1.097/2.762/0.638
Taylor Skill Score (TSS)	ADDA_V2	0.995/0.985/0.983	0.9990.999/0.658	0.995/0.999/0.425	0.986/0.999/0.552
	ERA5	0.951/0.999/0.436	0.977/0.959/0.667	0.972/0.959/0.661	0.968/0.981/0.629

*AK indicates Alaska and PR represents Puerto Rico.

As described in the Methods section, TSS depends upon spatial standard deviations. An overestimate of precipitation heterogeneity in ADDA_V2, possibly due to high spatial resolution, can result in higher spatial variabilities and lower TSS scores, even though ADDA_V2 demonstrates AV in many regions compared with ERA5.

In the CONUS, there are relatively large biases over the West Coast and Cascade Mountains during fall–spring, and the Southeast in the summer. However, ADDA_V2’s AV in those regions suggests it better represents mean daily precipitation over its forcing data. Compared to ERA5 reanalysis, ADDA_V2 better represents the location and intensity of the fall–spring heavy precipitation along the western coastline of the CONUS and over the Cascade and Sierra Nevada Mountains (Figs. 2 and S1). This improvement could be due to ADDA_V2’s ability to realistically resolve orography and orographically driven precipitation (more detailed discussions are provided in the supplementary information).

A noticeable dipole pattern of precipitation biases (i.e., a wet bias in the eastern Rocky Mountains and a dry bias in the central CONUS) is presented in Liu et al.^25^ is not present in summer and fall in our simulation. This is likely due to our simulation’s enhancements in realistically representing mountainous convection and the eastward propagation of associated systems. This, in turn, improves precipitation modeled in downstream regions, such as central and midwestern CONUS. This hypothesis is further discussed later in “Diurnal cycle of summer mean over the CONUS.”

Furthermore, ADDA_V2 better represents summer precipitation in the Southeast, particularly in Florida (Fig. 2i). Prior studies emphasized the significance of local environmental conditions and processes in summer total precipitation (e.g., sea-breeze in mesoscale convective systems and isolated storm development in the Southeast^35,36^). Florida experiences the most intense summer precipitation in the CONUS due to a distinctive process known as “cumulus-merger,”^37^ which is caused by its unique geographical location—surrounded by the ocean on three sides. During summer, peak precipitation occurs in the afternoon due to cumulus-merger (the convergence of sea breezes from the east and west coasts of the peninsula), causing strong convection over the peninsula. The improved representation of summer precipitation in Florida might be due to an enhanced representation of such local circulations. This process is further discussed in more detail in “Diurnal cycle of summer mean precipitation over the CONUS.”

For a more comprehensive examination, we used the probability density function to assess the precipitation distribution over the CONUS, Alaska, and Puerto Rico (Fig. 3) and the seven CONUS subregions (see Figs. 1b and S2). ADDA_V2 generally outperforms the ERA5 reanalysis, better capturing the overall distribution of mean daily precipitation (Fig. 3 and Table S2). In particular, for the CONUS, ADDA_V2 reasonably captures two observed distinct precipitation peaks in spring and fall. Table S2 demonstrates its superior performance in statistics, including average and variance, although ERA5 exhibits better skewness during these seasons. ADDA_V2 also better represents precipitation in intense ranges during winter. During summer, PRISM shows a more spread-out distribution of precipitation over the CONUS. ADDA_V2 not only simulates this distribution relatively well, but also better captures the intensity of summer precipitation compared to ERA5 reanalysis, which is displayed in statistics (Table S2). This improvement is particularly notable in regions with intense precipitation, such as the Southeast (Figure S2; JJA mean in the Southeast). There are also important discrepancies between ADDA_V2 and observations. For example, ADDA_V2 overestimates daily precipitation across all intensity levels, leading to a distribution shift toward more intense ranges in the Northeast in winter and spring (see Figure S2 and associated discussion). Conversely, ADDA_V2 underestimates precipitation, causing a bias toward moderate-to-low precipitation in the Southeast in spring (see Figure S2 and associated discussion). Over Alaska, ADDA_V2 is comparable to ERA5 across all seasons; both are similar to Daymet observations, although ERA5 exhibits overall better performance in winter, while ADDA_V2 is closer to observations in summer (Fig. 3e–h and Table S2). In Puerto Rico, ADDA_V2 tends to overestimate both intense and light precipitation, showing larger variances than Daymet (Fig. 3h–k and Table S2). This likely results in a noticeable contrasting bias on the western and eastern sides of the island across all seasons, as described above (Fig. 2c,g,k,o). This contrasting bias is likely attributed to an overestimation of orographic uplift associated with local circulation, such as sea-breeze trade wind convergence in western Puerto Rico, and an underestimation of trade wind driven orographic lift in the eastern portion^38^. Nevertheless, ADDA_V2 demonstrates superior performance over ERA5, especially in capturing topographic effects on precipitation; the northern two-thirds of the island is wetter than the southern portion^38,39^. ERA5, hindered by its coarse resolution, faces limitations in representing such intricate features compared to observation, leading to considerably narrower variances (Table S2).

### Diurnal cycle of summer mean precipitation over the CONUS

Recent studies have shown promising advances in representing the diurnal cycle of precipitation by explicitly resolving convection at CP resolution, providing notable AV over GCMs and convection-parameterized RCMs (e.g.,^14,40,41^). Here we focus on the June–August period because the diurnal pattern of precipitation is typically more pronounced during summer, and it can reflect the propagation of convective systems. For example, prior modeling studies documented that the failure of convection-parameterization can cause early onset of convection, increasing bias in timing and intensity of precipitation in the mid-to-late afternoon (e.g.,^42^).

**Figure 4:**
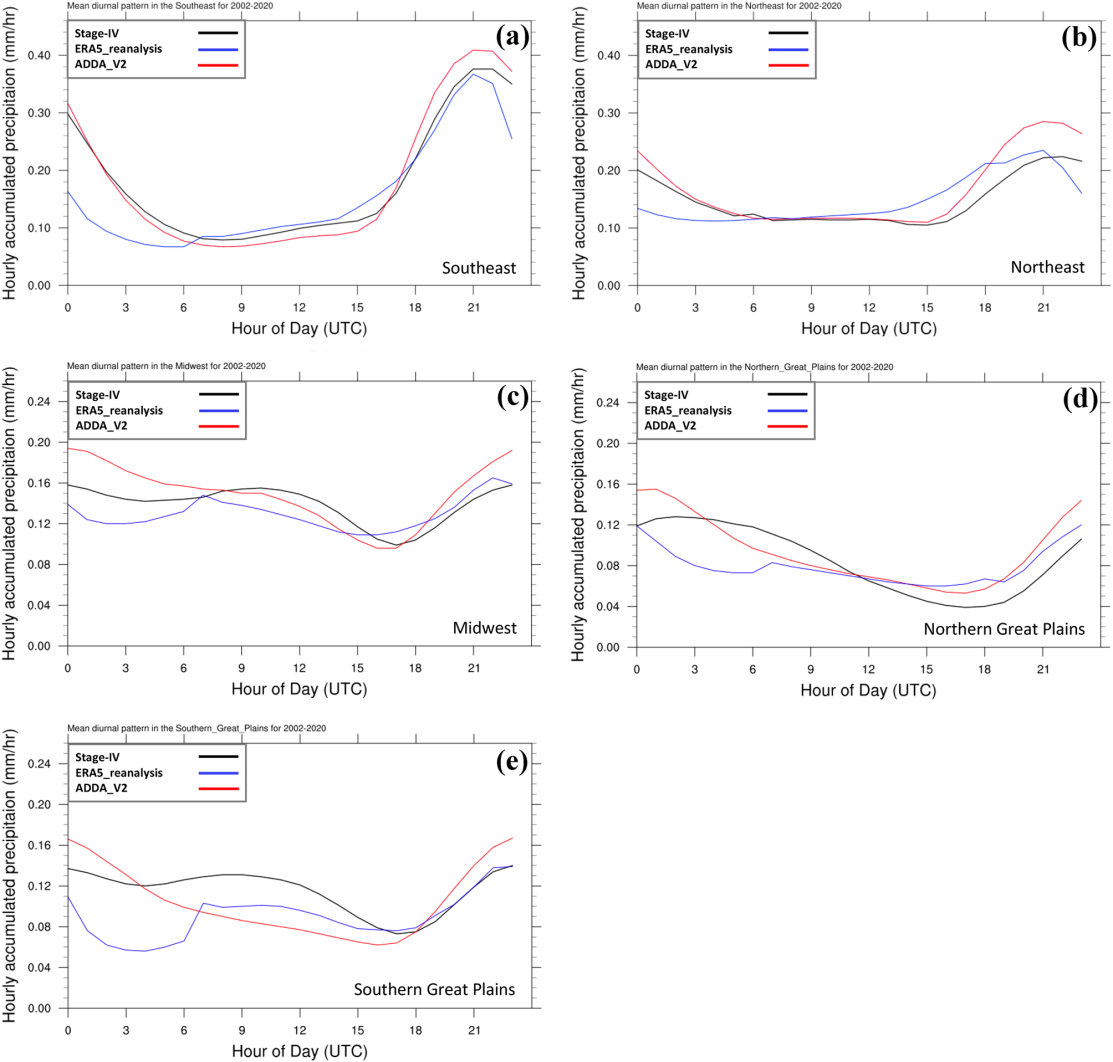
Mean diurnal cycle of hourly accumulated precipitation (mm hr^−1^) area-averaged over the five NCA subregions for JJA for the period of 2001–2020.

Figure 4 illustrates the summer mean diurnal cycle of hourly accumulated precipitation over the fourth National Climate Assessment CONUS subregions, as presented in Fig. 1b for ADDA_V2, compared to ERA5 reanalysis and NCEP Stage IV analysis (Stage IV hereafter). Here, we excluded the Northwest and Southwest due to a known issue in Stage IV in these regions (see “Datasets for evaluation” for more details). ADDA_V2 outperforms its forcing data (ERA5) over all subregions in terms of temporal pattern correlation and root mean square error (RMSE) of the precipitation diurnal cycle (Fig. 4; Table 3) because it reasonably captures the timing and variation of mid-to-late afternoon precipitation (local time) in all five subregions, compared to Stage IV. However, note that the ADDA_V2 tends to overestimate overall afternoon precipitation, especially over complex terrains (Fig. 4), indicating the model may overestimate the duration and intensity of precipitation events.

**Table 3:** PCC and RMSE of the JJA mean diurnal pattern of hourly precipitation averaged over the five NCA subregions for the period of 2002–2020.*

Metrics/data		Northeast	Southeast	Midwest	Northern great plains	Southern great plains
Patt. Corr.	ADDA_V2	0.98	0.99	0.83	0.84	0.63
	ERA5	0.63	0.88	0.62	0.60	0.34
RMSE	ADDA_V2	0.03	0.02	0.02	0.02	0.03
	ERA5	0.03	0.05	0.02	0.03	0.04

*PCC and RMSE are computed for ADDA_V2 and ERA5 reanalysis against Stage IV.

**Figure 5:**
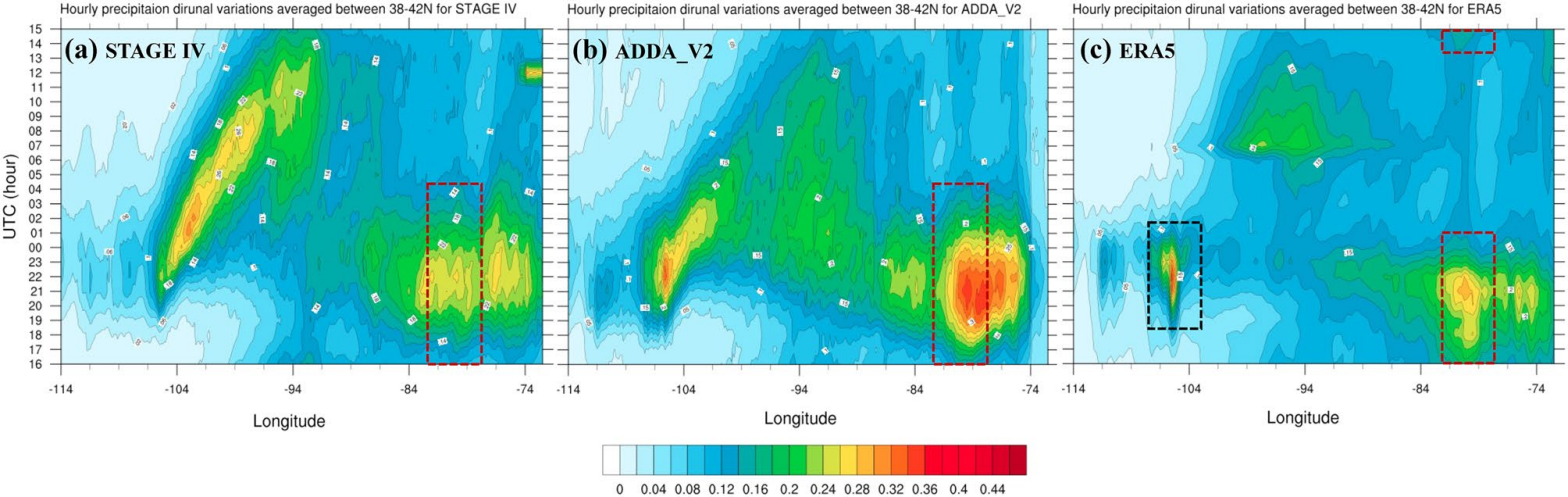
Hovmöller diagram of JJA mean hourly accumulated precipitation (mm hr^−1^) diurnal variations averaged between 38 and 42$^\circ$N for the period of 2002–2020 for (**a**) Stage IV, (**b**) ADDA_V2, and (**c**) ERA5 reanalysis. Red dashed boxes timings onset and dissipation of relatively intense precipitation (> 0.14 mm day^−1^) over the Appalachian plateau. Black dashed box indicates timings of the onset and dissipation of mountainous convection organized over the Rocky Mountains.

**Figure 6:**
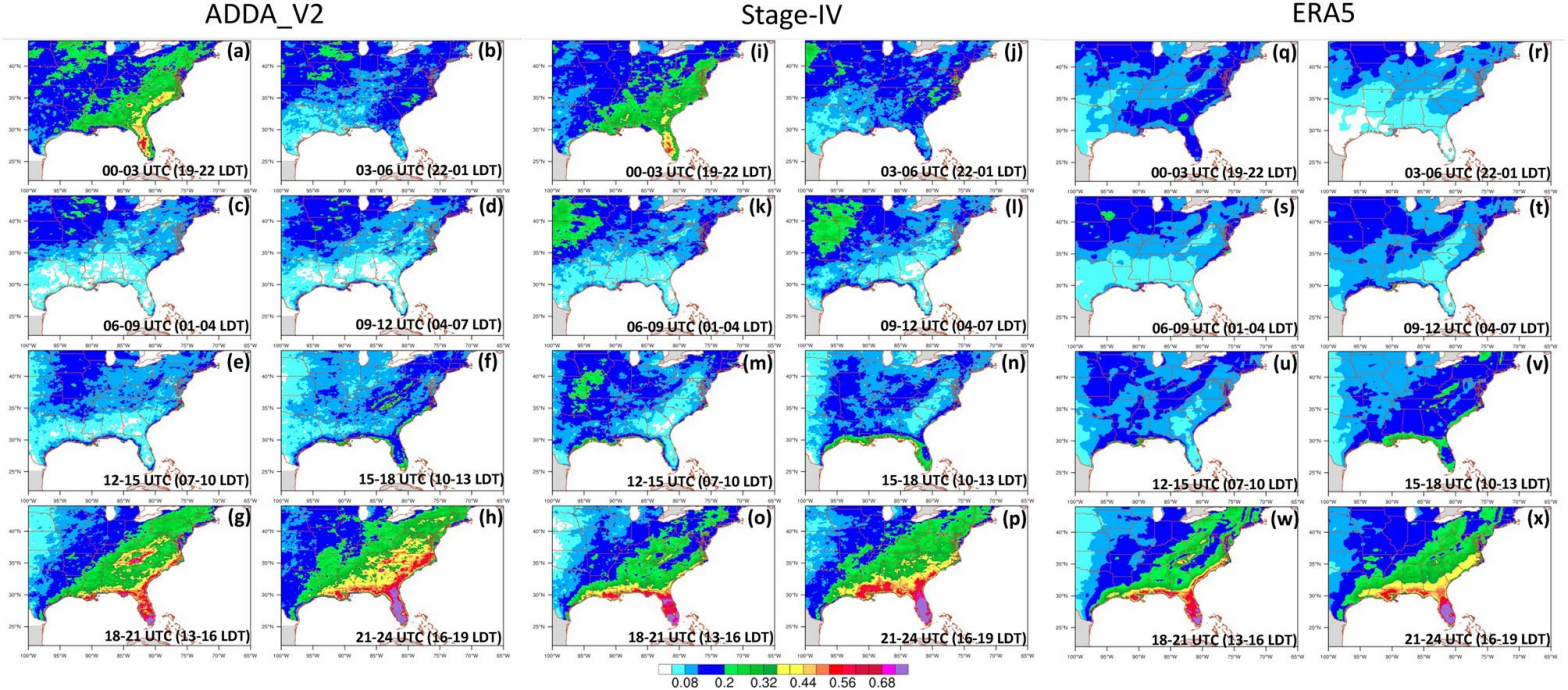
JJA diurnal cycle of hourly precipitation (mm hr^−1^) for (**a**)–(**h**) ADDA_V2, (**i**)–(**p**) Stage IV, and (**q**)–(**x**) ERA5 reanalysis for the period of 2002–2020. LDT indicates U.S. Eastern Time (i.e., local daylight time), which is 5 h behind than the Coordinated Universal Time (UTC). Subfigures display 3-hourly average precipitation rate (mm hr^−1^) during 00–03, 03–06, 06–09, 09–12, 12–15, 15–18, 18–21, and 21–00 UTC, respectively.

We further examine the process underlying ADDA_V2’s improved representation of summer precipitation by using the Hovmöller diagram (Fig. 5) and spatial diurnal cycle distribution (Fig. 6). Our focus extends from the Rocky Mountains and the Great Plains to the East Coast (zonally averaged area between 38 and 42$^\circ$N) in the Hovmöller diagram and encompasses the Southeast where ADDA_V2 outperforms ERA5 in summer precipitation in the spatial diurnal cycle distribution. As previously discussed, ADDA_V2 adds value in summer precipitation compared to ERA5 reanalysis, especially in the eastern half of the CONUS (Fig. 2i, hatched areas). ERA5 reanalysis broadly underestimates summer precipitation in the central and eastern CONUS, while a wet bias is obvious in the eastern Rocky Mountains (Fig. 2l). This suggests that there may be stationary mountain-generated convection that dissipates near its origin, often failing to form mesoscale convective systems that propagate off the Rocky Mountains to areas such as the Great Plains (e.g.,^25,43^).

To test this hypothesis, we use the Hovmöller diagram of the diurnal cycle of summer mean precipitation averaged between 38 and 42$^\circ$N in June–August 2002–2020 (Fig. 5). Figure 5 clearly depicts how different summer convective systems are presented in ADDA_V2 and ERA reanalysis. As we hypothesized, in ERA5 reanalysis, there is strong stationary mountainous convection organized over the Rocky Mountains in the late afternoon (local time ; Fig. 5c, black dashed box). The system fails to propagate downstream due to early dissipation near its origin. This causes precipitation to be underestimated overall downstream, as we discussed (Fig. 5c). Near the Appalachian plateau (8$2-$76$^\circ$W), ERA5 reanalysis reveals early onset and dissipation of relatively intense precipitation (> 0.14 mm day^−1^) around 13 and 02 UTC, respectively (Fig. 5c, red dashed boxes). These timings are approximately 3 h earlier than in Stage IV and ADDA_V2.

ADDA_V2 better represents the eastward propagation of mountainous convection that originates over the Rockies. This improves simulations of precipitation in downstream regions. In addition, ADDA_V2 captures the onset and dissipation of relatively intense precipitation (> 0.14 mm day^−1^) around 16 and 05 UTC, respectively, in the Appalachian plateau; these timings closely align with the observation from Stage IV despite an overestimation of peak intensity (Fig. 5a,b, red dashed boxes). ADDA_V2 also realistically simulates the suppression of convection over the Great Plains (104$-$98$^\circ$W) from afternoon to early evening (local time: 16–21 UTC). This suppression is likely caused by the downward return flow of the upslope wind in the upstream Rockies.

Tian et al.^44^ argued that the suppression of afternoon convection combines with the nighttime arrival of eastward-migrating convective storms generated the previous afternoon over the Rocky Mountains to produce precipitation that reaches its maxima near midnight over the Great Plains. This phenomenon is clearly illustrated in Figs. 4d and 5a. Specifically, over the Great Plains, diurnal precipitation peaks occur during late night and early morning hours, around 03–10 UTC.

As discussed, ADDA_V2 captures both the suppression of afternoon convection and the eastward propagation of the mountainous convective system better than the ERA5 reanalysis. However, the simulated mountainous convective system greatly decreases in intensity as it migrates east, which produces biases in downstream regions such as the northern Great Plains and Midwest. This early weakening of the system may contribute to early peaks in the simulated diurnal precipitation over the northern and southern Great Plains, as depicted in Fig. 4d–e. These findings suggest that topography and its associated impact on weather systems play a significant role in modulating the diurnal cycle of precipitation in these regions.

ADDA_V2 also shows a tendency to produce overly intense daily precipitation over complex terrains, such as the Rocky Mountains (104$-$106$^\circ$W) and Appalachian plateau (81$-$76$^\circ$W), which causes the maximum diurnal precipitation in the Northeast to be overestimated (Figs. 4b and 5b). This could be due to several factors, such as observational uncertainties (e.g.,^45,46^) or the limitations of our simulation’s horizontal grid spacing, which may not be fine enough to accurately capture the heterogeneity of the complex terrains. It may also depend on the representation of atmosphere–groundwater coupling, which plays an important role in evapotransportation and thus precipitation, as noted in Barlage et al.^47^.

On the other hand, in the coastal area of the Southeast, a robust diurnal cycle is present and is associated with local circulation (i.e., sea-breeze, resulting in a strong diurnal pattern of precipitation during summer). The diurnal precipitation distribution is presented in Fig. 6; ADDA_V2, Stage IV, and ERA5 all show the afternoon intensification of precipitation and its nighttime dissipation in regions such as Florida, the Gulf Coast, and the East Coast. However, when we focus on Florida alone, ERA5 reanalysis does not accurately depict the timing of precipitation dissipation (Fig. 6). More specifically, intense precipitation (> 0.4 mm/hr) is absent from the Florida peninsula during the late evening hours (19–22 LDT); however, both ADDA_V2 and Stage-IV capture this distinctive feature well (Fig. 6a,i,q). This finding suggests that ADDA_V2 reasonably represents local circulation and subsequent physical processes (e.g., sea-breeze convergence, cumulus-merger) taking place in the Florida peninsula, which leads to an enhancement in representing precipitation in the region over its forcing data (ERA5).

### The 95^th^ percentile and extreme precipitation

**Figure 7:**
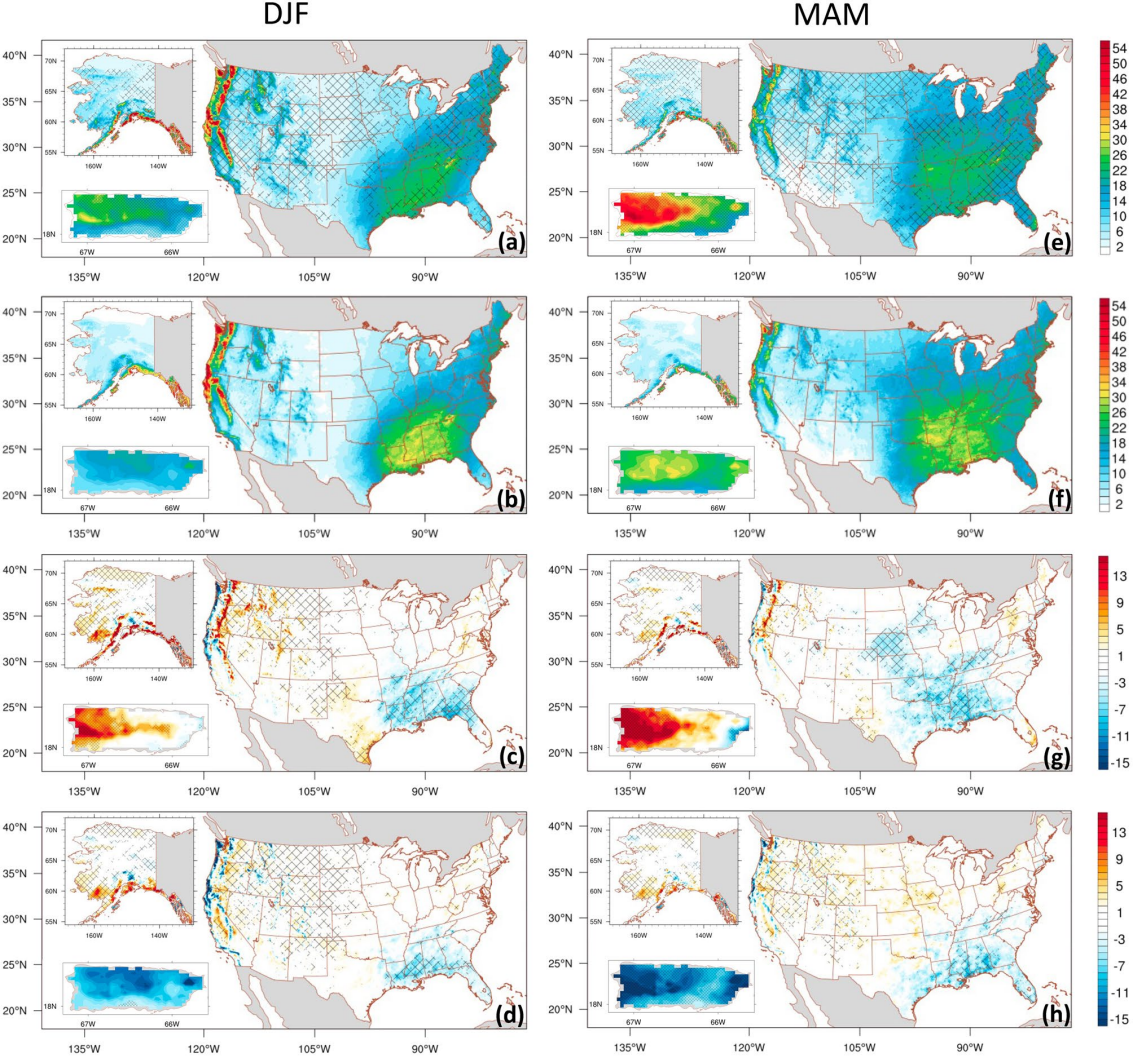

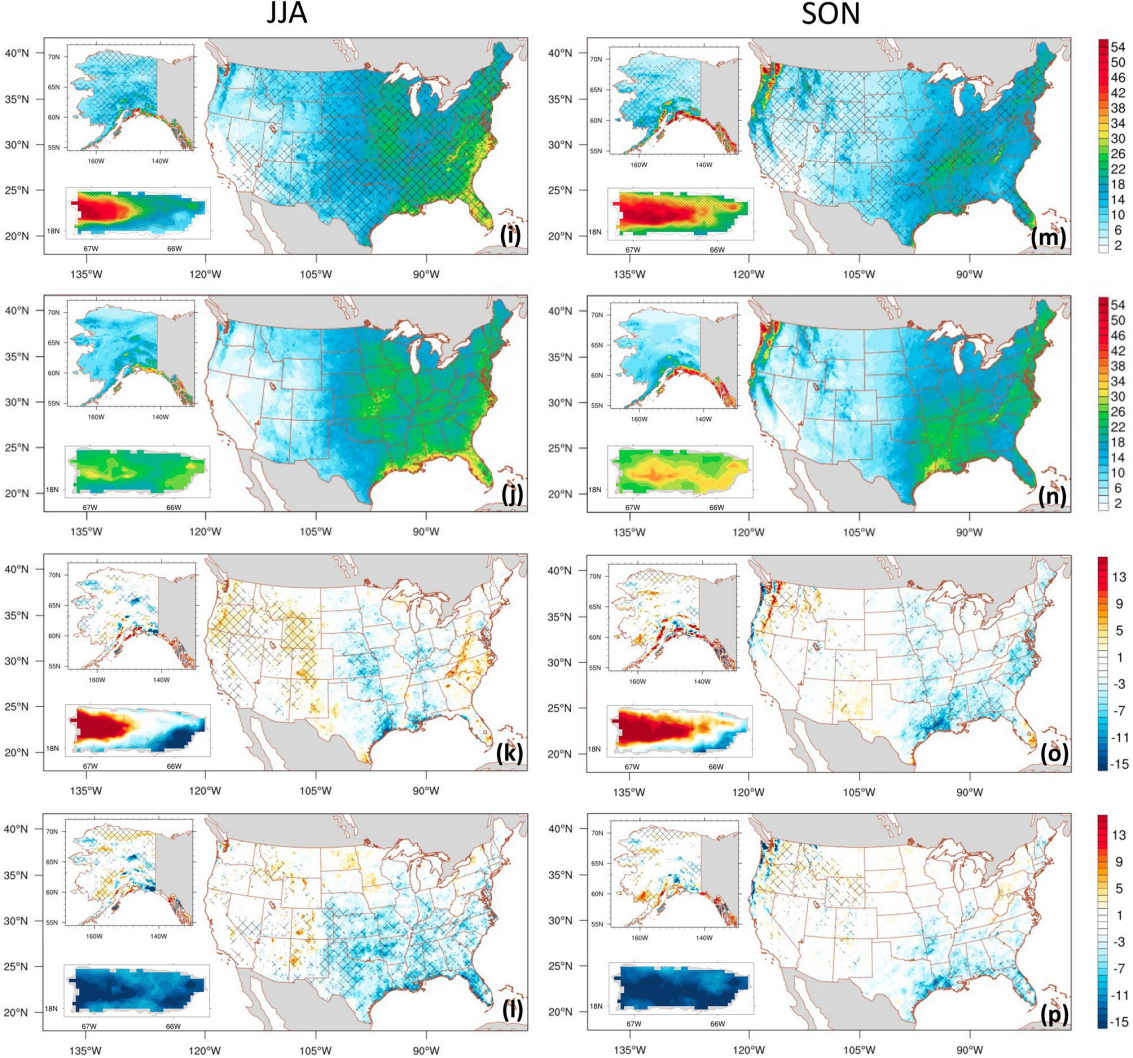
Spatial distribution of seasonal 95th percentile of daily precipitation (mm/day) for the CONUS, Alaska, and Puerto Rico during the 2001–2020 period. The data is sourced from ADDA_V2 (first row), Observation-based gridded dataset (second row), ADDA_V2 minus Observation-based gridded dataset (third row), and ERA5 minus Observation-based gridded dataset (fourth row). PRISM (Daymet) is utilized for CONUS (Alaska and Puerto Rico) as the observation-based gridded dataset. Hatches on the first row indicate grid points with value added by dynamical downscaling. In the third and fourth rows, grid points with statistically significant differences at 95% confidence level are marked with hatches.

To evaluate the model’s ability to capture intense precipitation, we investigate the 95th percentile of daily precipitation across all seasons. Results are presented in Fig. 7. Similar to the distribution of seasonal mean daily precipitation, the heaviest 95^th^ percentile precipitation is concentrated in the Southeast and Pacific Northwest CONUS, and southern Alaska. Mountainous areas spanning the middle of Puerto Rico also exhibit this pattern, which varies by season, as presented in Fig. 7 (second row).

Over the CONUS, both ERA5 (Fig. 7; fourth row) and ADDA_V2 (Fig. 7; first and third rows) capture the spatial pattern of 95th percentile precipitation. However, they both underestimate precipitation in many parts of the Southeast, West Coast, and Central United States compared to observations across all seasons. PRISM indicates that the magnitude of 95th percentile precipitation in the spring and fall is considerably lower than that in the winter; ADDA_V2 reproduces these spatial distributions, with improvements observed in hatched areas as depicted in Fig. 7a,e,m. There, AVs are 42.8%, 50.5%, and 48.4% for fall, spring, and winter, respectively. In summer, intense 95th percentile precipitation centers dominate the southeastern CONUS, especially along the coastlines. No intense precipitation centers are visible in the Northwest or Southwest in PRISM. Compared to ERA5 reanalysis, which considerably underestimates 95^th^ percentile precipitation with a spatial-averaged absolute bias of 4.66 mm day^−1^ over the Southeast, ADDA_V2 markedly reduces this bias in this region, yielding a spatial-averaged absolute bias of 0.67 mm day^−1^. Over Puerto Rico, ERA5 (ADDA_V2) grossly underestimated (overestimated) 95th percentile precipitation over the entire island (western half of the island) throughout all seasons. The bias exceeded ± 12 mm day^−1^, with a maximum dry bias of 65.4% and wet bias of 26.3% in ERA5 and ADDA_V2, respectively (Fig. 7, third and fourth rows). Over Alaska, the representation of 95th percentile precipitation by ERA5 and ADDA_V2 is quite robust; however, there is still a notable bias that is spatially consistent in both datasets. For instance, both ERA5 and ADDA_V2 overestimate 95th percentile precipitation over the southern coast of Alaska, with the bias more pronounced in the winter (11.9% overestimation for ERA5 and 27.8% for ADDA_V2) and fall (15.4% overestimation for ERA5 and 15.2% for ADDA_V2). Nevertheless, based on AV analysis, ADDA_V2 improves noticeably over the driving ERA5 in many grid points across all seasons in all three regions: up to 53.8%, 57.2%, and 85.6% of the total grid points in the CONUS, Alaska, and Puerto Rico, respectively.

However, note that ERA5 performance is better than ADDA_V2 over some grid points or regions. The improved representation of summer heavy precipitation by ADDA_V2, especially over the southeastern CONUS, may be due to improved simulation of local circulations and their associated processes, as discussed in “Summer mean diurnal precipitation over the CONUS,” above. This result may provide insights for further research in the field; improving heavy precipitation in climate models is crucial for effective flood management and water resource planning.

**Figure 8:**
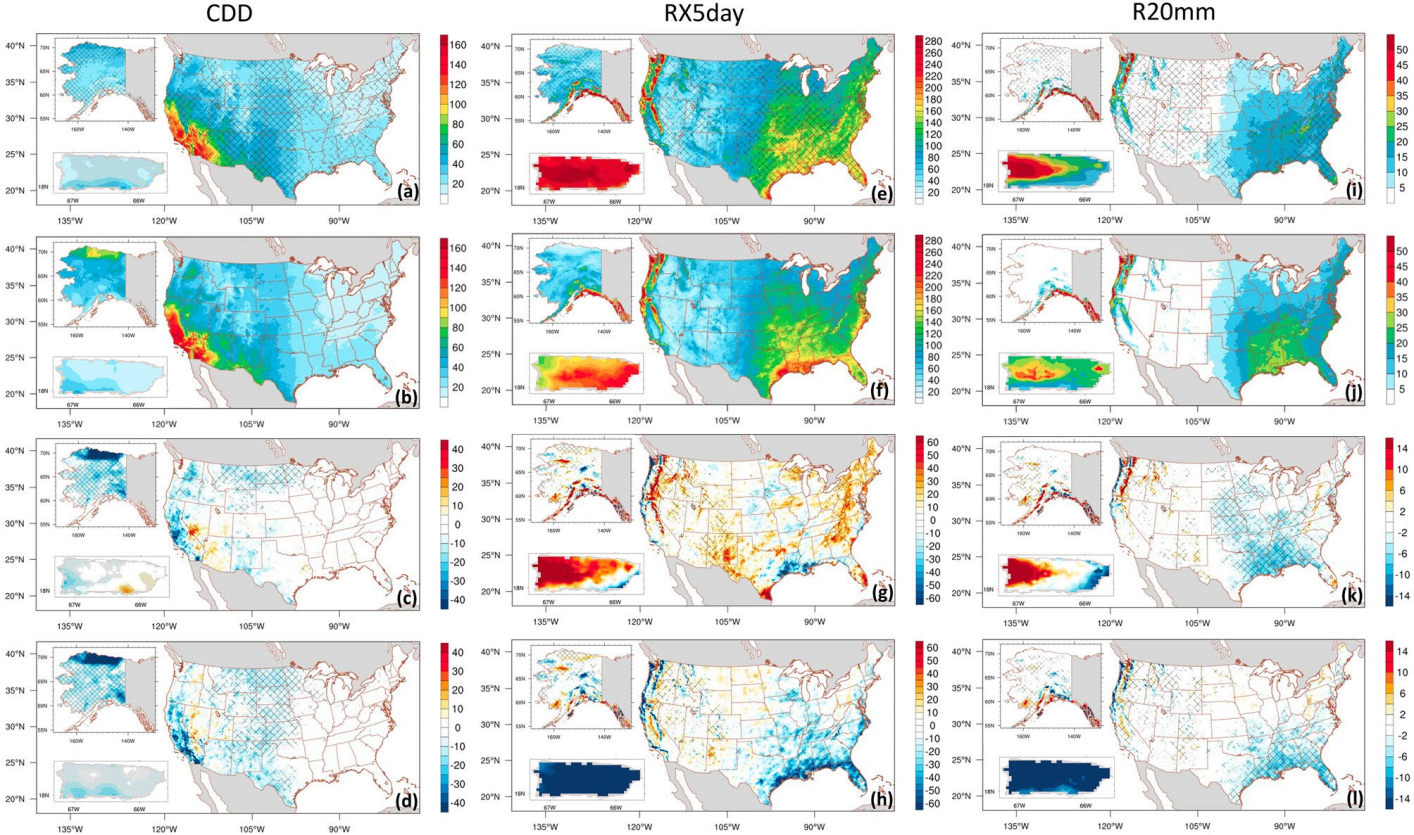
Spatial distribution of annual CDDs (first column; days), RX5day (second column; mm), and R20mm (third column; days) for the period of 2001–2020 for ADDA_V2 (first row), PRISM (second row), ADDA_V2 minus PRISM (third row), ERA5 reanalysis minus PRISM (fourth row). Cross-hatches in the first row indicate grid points with value added by dynamical downscaling. In the third and fourth rows, grid points with statistically significant differences at 95% confidence level are marked with hatches.

In addition, we evaluate the spatial distribution of the three extreme indices: annual mean consecutive dry days (CDDs, number of consecutive days with precipitation < 1 mm), maximum five-consecutive-day precipitation (RX5day), and very heavy precipitation days (R20mm) defined in Table 1. Results are presented in Fig. 8. These indices have been extensively used to indirectly assess the potential occurrence of drought and flood events in many regions.

Over the CONUS, as in observations, the Southwest has the most CDDs (> 140 per year) and the Northeast and Midwest have the fewest CDDs (< 20 per year) (Fig. 8b). Over Alaska, the minimum (maximum) CDD occurs over the southern part (northern part) of the state. Values range from 10 to 100 days. In Puerto Rico, most regions experience fewer than 20 CDDs, and southwestern areas have 30–40 dry days.

Relative to the observations, the ADDA_V2 realistically reproduces the spatial pattern of CDDs. It captures regions of maximum and minimum values across all three regions, although a noticeable bias still exists. For instance, ADDA_V2 underestimated CDD over most of Alaska, northern and western/southwestern Puerto Rico, and southwestern CONUS (Fig. 8a,c). This indicates that it produces more wet days, consistent with the wet bias over these regions.

However, in comparison to ERA5 reanalysis (Fig. 8d), ADDA_V2 improves slightly by reducing the spatial bias over several grid points. This improvement is clearly evident in the hatched areas in Fig. 8a, primarily across the northern and southern Great Plains, southwestern CONUS, wide areas of Alaska (excluding the middle region), and Puerto Rico (except the western part of the island). ADDA_V2 demonstrates improvement over about 60.0% of all grid points in the CONUS, 66.8% in Alaska, and 74.1% in Puerto Rico (Fig. 8a).

For the RX5day, the maximum center seen in the observations primarily occurs over the southeastern and northwestern CONUS, mountainous areas in the middle and northeastern part of Puerto Rico, and southern parts of Alaska, with values reaching 240 mm (Fig. 8f). In contrast, the state of Nevada, western Puerto Rico, and northern Alaska experience the minimum values, which do not exceed 100 mm. Relative to observations, ERA5 reanalysis (Fig. 8h) captures the spatial pattern over CONUS and Alaska. However, the magnitude is considerably lower in the eastern half of the CONUS, resulting in a pronounced underestimation over the Southeast, the West Coast, and the Cascade and Sierra-Nevada Mountains. Similarly, ERA5 grossly underestimates RX5day, with a spatial-averaged bias of 45.2% over the entire region of Puerto Rico. ADDA_V2, on the other hand (Fig. 8e,g), reasonably captures the observed pattern. However, it tends to overestimate RX5day in the Northeast, Cascade Mountains, western parts of the southern Great Plains by up to 21.3% in the regions, and most of Puerto Rico with a spatial-average bias of 24.6% across the island; it underestimates RX5day along the West Coast and Gulf Coast near Texas and Louisiana. Nevertheless, ADDA_V2 demonstrates improvement over about 49% of all grid points in the CONUS, 51.2% in Alaska, and 80.8% in Puerto Rico (Fig. 8e).

Over the CONUS, the R20mm is greatest (smallest) over the Southeast, the West Coast, and the Cascade Mountains (the western half of the United States); values reach 25 days or more per year (Fig. 8j). Heavy R20mm is most frequent over southern coastal Alaska, central parts of the western half of Puerto Rico, and Fajardo (Fig. 7j). ERA5 reanalysis reproduces the observed distribution of R20mm over the CONUS and Alaska (Fig. 8l), but it significantly underestimates the magnitude of R20mm over the northwestern and southeastern CONUS and overestimates it over coastal Alaska. Similar to daily mean precipitation and RX5day, ERA5 grossly underestimates R20mm over all of Puerto Rico, with a spatial-average bias of 43.2% across the island. ADDA_V2, on the other hand, shows lower bias in R20mm over central Puerto Rico, the northern and southern Great Plans, and central-northern Alaska (Fig. 8i,k). However, ADDA_V2 grossly overestimates R20mm by 40.2% over the western half of Puerto Rico and underestimates it by 43.8% over the eastern half. Overall, ADDA_V2 improves on the ERA5 results across more than 44.7% of all grid points in the CONUS (mostly over the western half and East Coast), 53.4% in Alaska, and 93.2% in Puerto Rico.

## Summary and conclusions

In this study, we assess a 20-year dynamically downscaled climate simulation at 4-km CP resolution across the CONUS, Alaska, and Puerto Rico. We evaluate its performance in representing mean and heavy precipitation characteristics across time scales in these regions during 2001–2020. In addition to comparing the results with high-resolution PRISM over the CONUS and with Daymet over Alaska and Puerto Rico, we explore the AV of the CP simulation in reproducing mean and heavy precipitation, and discuss the potential processes that may contribute to this AV.

Our findings reveal that, compared with forcing data from ERA5 reanalysis, CP simulation with explicit convection improves representations of seasonal mean precipitation over a large portion of the CONUS, Alaska, and Puerto Rico, particularly in the areas where precipitation is heaviest. Overall, the simulation better captures the 95th percentile and extreme indices, such as CDD, RX5day, and R20mm across the three regions and seasons, exhibiting greater consistency with PRISM and Daymet. Also, note that ERA5 results are better than ADDA_V2 in some instances.

When evaluating summer mean hourly precipitation, ADDA_V2 has the following added values compared to ERA5 analysis: (1) improved representation of precipitation intensity at hourly time scales; (2) accurate timing (onset and peak) of the diurnal cycle of summer precipitation; (3) better representation of the eastward-propagating convective precipitation that originates over the Rockies, which produces better simulations of downstream precipitation; (4) a more accurate depiction of the downward return flow of upslope wind in the Rockies, which produces better representations of the daytime suppression of convection over downstream regions (i.e., the Great Plains); and (5) realistic representations of local circulation and subsequent physical processes (e.g., sea-breeze convergence, cumulus-merger) over Florida.

Our findings align with previous studies that employed a CP approach for various regions^20,25,26,48,49^. This consistency highlights the benefits of using CP scale to accurately represent seasonal mean and extreme precipitation. It enhances confidence in the potential for studying climate change and its impact assessment utilizing CP simulations. The quantitative bias and bias distribution for ADDA_V2 reported herein will provide WRF model developers with a roadmap for needed model improvements. It also offers valuable insights to guide the design of future model experiments aimed at enhancing the accuracy of local and regional-scale precipitation projections in a warming climate.

## Methods

### Model description

In this study, we used the Weather and Research Forecasting (WRF) version 4.2.1^50^ to examine a single domain of 2050 × 1750 horizontal grid points (8200 km × 7000 km) at 4-km grid spacing. This domain has more than 1.79 million grid cells, which cover almost all of North America and the Caribbean islands, including Puerto Rico (Fig. 1a). In the vertical, 50 unevenly spaced σ levels from the surface up to 50 hPa with 18 σ levels below 1 km and approximately 200 m resolution in the upper troposphere^51^. The model featured explicit convection, the Morrison microphysics^52^, the Yonsei University (YSU) planetary boundary layer^53^, the rapid radiative transfer model (RRTMG^54^) for long and short wave radiations, and the Unified Noah land-surface model^55^. Single-domain model simulations were integrated with output saved every 1 h.

We did not employ any convective parameterization, because previous studies have documented that clouds and deep convection can be reasonably resolved at a spatial resolution of 4 km or higher (e.g.,^25,56–59^). The initial and lateral boundary conditions are specified by the European Centre for Medium-Range Weather Forecast reanalysis product (ERA5^32^) for a period from 2001 to 2020. We use five variables at 37 pressure levels (i.e., geopotential, temperature, meridional and zonal wind vectors, relative humidity) and 26 single-level variables (e.g., 2-m temperature, 10-m meridional and zonal wind vectors, surface pressure), as outlined in Table S1, to provide initial and lateral boundary conditions. The ocean and lake temperatures were prescribed to be the same as the ERA5, and updated every 6 h. The one-dimensional lake model available in WRF was not implemented in this study.

In accordance with prior studies^15,60–63^, a series of 14-month runs with 20 reinitializations were performed. That is, rather than running the simulations continuously for 20 years, the model is initialized on November 1st of the previous year and is continued all the way to the end of the current year. The applicability of reinitialization in long-term simulations is discussed in more detail in the supplementary information.

To minimize imbalances and adjustment issues that arise from the reinitialization of each year, a 2-month spin-up period (November and December) is excluded from analysis in this study. No internal grid nudging or spectral nudging technique is applied, so that the model can develop its own variability (e.g., spatial and internal variability) across the domain. The output data includes hourly variables near the surface and in vertical profiles of the most frequently used variables (e.g., temperature, winds, moisture, pressure, precipitation, and geopotential). Other variables that are used less often, based on our previous experience, are output every 3 h.

The simulations were performed at the Argonne Leadership Computing Facility (ALCF) on the Theta cluster, using the computational power of 64-core, 1.3-GHz Intel Xeon Phi 7230 processors. The simulations required a total of 500,000 node hours and 6400 h of wall clock time to complete the 20-year simulation. This extensive simulation generated approximately 1.7 petabytes of data, which are stored in the ALCF’s high-performance storage system.

### Datasets for evaluation

The simulation was evaluated by focusing on comparing the CONUS, Alaska, and Puerto Rico against the high-resolution (4 km, daily) observation-based gridded dataset PRISM^33^; the National Centers for Environmental Prediction (NCEP) Stage IV hourly radar-gauge based precipitation product^64^; Daily Surface Weather Data on a 1-km Grid for North America, Version 4 (Daymet^34^); and the simulation’s forcing data, ERA 5 reanalysis^32^.

Daily aggregates of PRISM, Daymet, and ERA5 daily precipitation from 2001 to 2020 were used to compute the precipitation mean and extreme indices, including annual and seasonal (i.e., winter: December–January–February, DJF; spring: March–April–May, MAM; summer: June–July–August, JJA; fall: September–October–November, SON) mean values, the 95th percentile of precipitation, and three extreme indices defined by the Expert Team on Climate Change Detection and Indices (ETCCDI, Table 1). The ETCCDI includes CDDs, very heavy rainfall days (R20mm), and RX5day.

The process of creating the averages involved calculating the 95th percentile precipitation and three extreme indices for each individual year. Then, we computed their averages over the 20-year period. All the temporal averages were computed using the native resolution of each dataset.

NCEP Stage IV hourly data (mm hr^−1^) for the summer (i.e., June–August) from 2002 to 2020 were used to validate model–observation discrepancies in the diurnal pattern of precipitation and examine the intensity and propagation of convective systems that are initiated on the complex terrain of the Rocky Mountains. Note that NCEP Stage IV is available starting from 2002, and has issues over regions west of 114°W, according to Chang et al.^65^ and Nelson et al.^66^ Therefore, we only looked at regions east of 114°W for validation of diurnal pattern. For direct comparison between the 4-km simulation, PRISM, and ERA5, all the calculated statistics were regridded to a 0.25° × 0.25° resolution (the lowest ERA5 reanalysis resolution) by using bilinear interpolation. Despite aggregating high-resolution data to coarse resolution to match the reanalysis data, the high-resolution data still exhibit superior performance in capturing spatial features, compared to the low-resolution data (e.g.,^67^). Therefore, this method enables us to make a fair comparison between the three datasets, ADDA_V2, PRISM_4km, and ERA5_30km.

### Metrics for evaluation

The performance of ADDA_V2 is quantified based on a suit of statistical metrics presented below. It includes RMSE and pattern correlation coefficient (PCC), respectively:

$${\text{RMSE}}=\sqrt{\frac{1}{n}{\sum }_{i=1}^{n}{\left({M}_{i}-{O}_{i}\right)}^{2}}$$

$$\mathrm{PCC }\left(M,O\right)=\frac{\sum ({M}_{i}-\overline{M })({O}_{i}-\overline{O })}{\sqrt{\sum {({M}_{i}-\overline{M })}^{2}{({O}_{i}-\overline{O })}^{2}}}$$

where ${M}_{i}$ and ${O}_{i}$ are model and observation data at each point. $\overline{M }$ and $\overline{O }$ are model and observation means, respectively. The n represents the number of observations.

The model evaluation focuses on mean and 95th percentile precipitation and their spatial variabilities and magnitude over each grid cell. These later two aspects are important because even data with low spatial resolution can produce small RMSE with very smooth spatial patterns. High-spatial-resolution data, however, can produce high spatial variabilities that need to be measured in ways other than RMSE. Following Hirota et al.^68^, TSS^69^ was computed to evaluate the performance of the 4-km simulation in annual and seasonal mean precipitation over the CONUS. The skill score is defined as:

$$S\equiv \frac{{(1+{\text{PCC}})}^{4}}{{4\left({\text{SDR}}+\frac{1}{{\text{SDR}}}\right)}^{2}}$$

where PCC indicates the pattern correlation coefficient between the models and reference data and SDR is the ratio of the spatial standard deviations of the models against that of reference data. Therefore, this score measures how closely the spatial pattern and amplitude of the model match those of the observation. A score of 1 indicates a perfect match between the model and observation, while a value of 0 represents no skill in the model. The score is computed based on the PCC and SDR of the seasonal mean over the CONUS. RMSE, PCC, and the AV approach are also used to assess the performance of the 4-km simulation. The AV approach proposed by Di Luca et al.^70^ is designed to quantify the downscaled output performance compared with its coarse forcing data (ERA5). The AV is defined here according to Dosio et al.^71^ and Akinsanola and Zhou^72^:

$${\text{AV}}=\frac{{\left({X}_{{\text{ERA}}5}-{X}_{{\text{OBS}}}\right)}^{2}-{\left({X}_{{\text{ADDA}}\_{\text{V}}2}-{X}_{{\text{OBS}}}\right)}^{2}}{{\text{Max}}\left({\left({X}_{{\text{ERA}}5}-{X}_{{\text{OBS}}}\right)}^{2},{\left({X}_{{\text{ADDA}}\_{\text{V}}2}-{X}_{{\text{OBS}}}\right)}^{2}\right)}$$

where *X*_ERA5_, *X*_OBS_, and *X*_ADDA_V2_ indicate values from ERA5 (forcing data), observation (PRISM/Daymet), and ADDA_V2. The value falls within the range of − 1 to 1, based on prior work^70^ and is computed on every grid cell. AV becomes positive when the squared error of the 4-km simulation is smaller than that of the corresponding ERA5 reanalysis, indicating that the 4-km model generates results that are closer to the observations compared to ERA5. AV indicates the percentage of grid cells that show improvement out of the total grid cells.

### Supplementary Information

Supplementary Information.

## Data Availability

All datasets used in this study are freely available. ERA5 reanalysis data are publicly available through Climate Data Store: https://cds.climate.copernicus.eu/. PRISM data are obtained from https://prism.oregonstate.edu/. Stage IV data are retrieved from Earth Observing Laboratory: https://data.eol.ucar.edu/dataset/21.093. The ADDA V2 data generated for the study are located on the ALCF high-performance storage system and are being uploaded to the Climate Risk & Resilience Portal (https://disgeoportal.egs.anl.gov/ClimRR/) for public use.
