# Supplementary material for: Evaluation of precipitation across the contiguous United States, Alaska, and Puerto Rico in multi-decadal convection-permitting simulations
Source: Sci Rep. 2024 Jan 12;14:1238. doi: 10.1038/s41598-024-51714-3 (PMC10786863; doi:10.1038/s41598-024-51714-3)
Supplement: Supplementary file 1 — Supplementary Information. [file 41598_2024_51714_MOESM1_ESM.docx]

**Evaluation of Precipitation across the Contiguous United States, Alaska, and Puerto Rico in Multi-decadal Convection-Permitting Simulations**

 Akintomide Afolayan Akinsanola^1,2^, Chunyong Jung^1^**^*^**, Jiali Wang^1^, and Veerabhadra Rao Kotamarthi^1^

^1^Environmental Science Division, Argonne National Laboratory, Lemont, IL, USA

^2^Department of Earth and Environmental Sciences, University of Illinois Chicago, Chicago, IL, USA

**(Supplementary Information)**

*Corresponding author address: Chunyong Jung, Environmental Science Division, Argonne National Laboratory, 9700 South Cass Avenue, Building 240, Lemont, IL, 60439.

E-mail: [cjung2@anl.gov](mailto:cjung2@anl.gov)

**1. Discussion on orographically driven precipitation along the western mountainous areas**

As discussed in the text, compared to ERA5 reanalysis, ADDA_V2 better represents the location and intensity of the fall–spring heavy precipitation along the western coastline of the CONUS and over the Cascade and Sierra Nevada Mountains. To further examine, we have added a figure displaying Hovmöllor diagram of 20-year mean monthly daily precipitation averaged between 35-50$^{\circ}$N for PRISM, ADDA_V2, and ERA5 (Figure S1). In the figure, ADDA_V2 exhibits an improved presentation of the location and intensity of the cool season (Oct-Apr) heavy precipitation along the western coastline (near 124$^{\circ}$W) and the Cascade and Sierra-Nevada Mountains (near 122$^{\circ}$W). Especially, during the period from November to February., the peak precipitation in the mountainous areas is concentrated along the longitude range of 121.8$^{\circ}$W to 122.3$^{\circ}$W, with an approximate rate of ~7.5 mm/day and ADDA_V2 appears to reasonably capture the location and intensity (although it slightly overestimates the intensity). On the other hand, the location of the peak precipitation over the mountainous regions in ERA5 shifts towards the west and noticeably underestimates the intensity compared with both PRISM and ADDA_V2. Given these are mountainous areas, the bias of ERA5 could be attributed to the coarse resolution of orography and vice versa for the improvement in ADDA_V2. In fact, we cannot confirm that the orographic lift is better represented in ADDA_V2 by analyzing precipitation only, but an improvement in precipitation over mountainous regions is likely driven by an improvement in resolving orography and thus orographically driven precipitation.

**2. Representation of mean precipitation in the seven U.S. subregions**

The ADDA_V2 generally demonstrates improved performance compared to the ERA5 reanalysis, reasonably capturing the variability of seasonal mean daily precipitation across the CONUS during all four seasons (Figure 3). However, since the seasonal PDFs presented in Figure 3 were derived by aggregating data from all grid cells across the CONUS, it is plausible that discrepancies in one region might counterbalance those in other regions. In other words, when errors in different regions have opposite effects on the overall distribution, they can offset each other when the data is plotted in a PDF. Therefore, conducting this analysis on regions that are more climatologically homogeneous is necessary to avoid this concern. The CONUS is divided into seven subregions according to the fifth National Climate Assessment subregions for this purpose (Figure 1b). The distribution and variability of seasonal mean daily precipitation simulated by ADDA_V2 generally matches its observational counterpart across the regions and seasons. In particular, ADDA_V2 provides a more accurate representation of the precipitation distribution in regions where the maximum precipitation occurs during winter (DJF; Northwest) and summer (JJA; Southeast). Further, consistent with the analysis in the manuscript, clear improvements in the summer precipitation distribution to the east of the Rockies, such as the Midwest and Southern Great Plains have been identified (Figure S2). These enhancements can be attributed to an improved representation of the eastward propagation of mountainous convection that originates in the Rockies as discussed in the manuscript. Nonetheless, there exist some considerable discrepancies as depicted in Figure S2. For example, ADDA_V2 demonstrates a large overestimation of daily precipitation across all intensity levels, leading to a distribution shift towards more intense ranges in the Northeast in DJF-MAM. Conversely, ADDA_V2 underestimates precipitation, causing a bias towards moderate-to-low precipitation ranges in the Southeast in MAM. It is worth noting that the improvements are not evident in the Northern Great Plains during JJA because the region contains a large portion of windward and mountainous areas of the Rockies where excessive shallow convections are observed, causing the overestimation of low intensity precipitation in the region (Figure S2).

**3. The applicability of reinitialization in long-term simulations**

In general, there are two strategies for dynamical climate downscaling. One approach involves a continuous long-term integration, while the other approach re-initializes the model frequently. While the first approach is currently a common practice for regional climate downscaling, concerns have arisen regarding the issue of systematic error accumulation, which can lead to drift from the forcing fields during long-term integration. To address this, recent dynamical climate downscaling studies have incorporated nudging techniques, aimed at preventing the divergence of synoptic-scale features within the regional climate model from global climate model lateral and boundary conditions, thereby mitigating the drift. The latter approach, i.e., model reinitialization, also minimizes possible drift caused by accumulated model errors. Additionally, it allows several segments of a long simulation to be run in parallel.

Several studies have compared these two approaches, assessed their respective model performances, and discussed the applicability of reinitialization in long-term simulations. For example, Qian et al. (2003) identified two distinct stages in the model spin-up process. In the initial day or two, the model rapidly adjusted to a state intrinsic to its dynamics, driven by the removal of small-scale imbalances through geostrophic adjustment from mass to wind field. Over the subsequent 15 to 20 days, the model variables gradually converged to a state closely resembling that of a long-term continuous run. At this point, the simulation results closely matched those of the continuous run. Similarly, Lucas-Picher et al. (2013) demonstrated the majority of small-scale variabilities and vortices were fully generated during this period. Furthermore, Pan et al. (1999) demonstrated that when examining the spatial and temporal distributions of various variables, such as rainfall, mixing ratio, temperature, and atmospheric forcing play a more crucial role than soil moisture. They drew this conclusion by conducting two experiments: one involving the reinitialization of all forcing fields and another in which only the atmospheric variables were reinitialized, while the soil moisture remained unchanged. These prior works collectively validate the feasibility and validity of subdividing long-term integrations into shorter ones.

**Table S1.** List of ERA5 variables used as input.

| S/N | Name | Units | S/N | Name | Units |
| --- | --- | --- | --- | --- | --- |
| 1 | U-component wind | m s^-1^ | 17 | Surface pressure | Pa |
| 2 | V-component wind | m s^-1^ | 18 | Soil temperature level 1 | K |
| 3 | Geopotential | m^2^ s^-2^ | 19 | Soil temperature level 2 | K |
| 4 | Relative humidity | % | 20 | Soil temperature level 3 | K |
| 5 | Temperature | K | 21 | Soil temperature level 4 | K |
| 6 | Sea-ice cover | (0-1) | 22 | Snow depth | m |
| 7 | Snow albedo | (0-1) | 23 | Snowfall | m |
| 8 | Snow density | kg m^-3^ | 24 | Mean sea level pressure | Pa |
| 9 | Sea surface temp. | K | 25 | 10-m U-component wind | m s^-1^ |
| 10 | Volumetric soil water layer 1 | m^3^ s^-3^ | 26 | 10-m V-component wind | m s^-1^ |
| 11 | Volumetric soil water layer 2 | m^3^ s^-3^ | 27 | 2-m temperature | K |
| 12 | Volumetric soil water layer 3 | m^3^ s^-3^ | 28 | Land-sea mask | (0-1) |
| 13 | Volumetric soil water layer 4 | m^3^ s^-3^ | 29 | Skin temperature | K |
| 14 | Soil type |  | 30 | Temperature of snow layer | K |
| 15 | Snow evaporation | m of water | 31 | Total column snow water | kg m^-3^ |
| 16 | Snow melt | m of water |  |  |  |

**Table S2.** Statistics for the probability density function of daily mean seasonal precipitation.

| **Statistic/data** | | **DJF**  **(CONUS/AK/PR)** | **MAM**  **(CONUS/AK/PR)** | **JJA**  **(CONUS/AK/PR)** | **SON**  **(CONUS/AK/PR)** |
| --- | --- | --- | --- | --- | --- |
| Average  (mm/day) | Obs. | 10.35 / 10.71 / 4.33 | 6.02 / 6.73 / 5.34 | 4.88 / 7.42 / 6.04 | 8.51 / 13.92 / 8.13 |
|  | ADDA_V2 | 10.84 / 15.00 / 3.30 | 7.45 / 10.12 / 6.46 | 5.49 / 9.18 / 9.67 | 8.64 / 16.57 / 8.97 |
|  | ERA5 | 6.24 / 7.53/ 1.54 | 3.78 / 5.12 / 2.78 | 4.01 / 5.99 / 3.33 | 4.92 / 8.94 / 4.33 |
| Variance  (mm/day) | Obs. | 35.67 / 38.69 / 3.81 | 12.07 / 15.12 / 3.18 | 8.11 / 15.69 / 3.73 | 24.17 / 63.79 / 3.74 |
|  | ADDA_V2 | 38.68 / 74.64 / 2.42 | 18.45 / 33.11 / 10.28 | 10.12 / 24.65 / 28.14 | 24.94 / 89.69 / 13.91 |
|  | ERA5 | 12.03 / 17.31 / 0.25 | 4.63 / 7.62 / 0.66 | 5.47 / 8.66 / 0.78 | 7.79 / 23.26 / 0.66 |
| Skewness  (${\times10}^{-6}$) | Obs. | 4.67 / 6.38 / -0.71 | 3.56 / -0.16 / 5.12 | 1.08 / 0.45 / -3.97 | -1.44 / -5.13 / 34.7 |
|  | ADDA_V2 | -5.91 / -3.35 / 3.86 | -8.96 / -1.79 / -3.23 | -3.97 / 0.69 / -3.99 | -9.13 / -7.49 / -13.5 |
|  | ERA5 | 1.70 / 0.59 / 2.63 | 1.43 / -4.83 / 2.51 | -2.78 / 1.13 / 1.47 | -1.47 / 1.26 / 3.44 |

* AK indicates Alaska and PR represents Puerto Rico.

Figure S1. Hovmöller diagram of 20-year mean monthly daily accumulated precipitation (mm day^-1^) variations averaged between 35 and 50°N for the period of 2001–2020 for (a) PRISM, (b) ADDA_V2, and (c) ERA5 reanalysis.

Figure S2. Probability density function (PDF) of seasonal mean daily precipitation (mm day-1) for the seven contiguous U.S. subregions presented in the fifth National Climate Assessment. Each region and season are presented in the plot’s upper right corner and bottom, respectively.

Figure S2*.* (*Continued*)

Figure S2*.* (*Continued*)

Figure S2*.* (*Continued*)
